# Cultural Adaptation and Psychometric Validation of the Simplified Chinese Version of the Fear Avoidance Component Scale (FACS)

**DOI:** 10.1155/prm/7966689

**Published:** 2024-12-05

**Authors:** Zhongyi Tu, Junfang Miao, Yanzhao Zhang, Zhaohui Yang, Rui Xu, Randy Neblett

**Affiliations:** ^1^Department of Rehabilitation Medicine, Union Hospital, Tongji Medical College of Huazhong University of Science and Technology, Wuhan 430023, China; ^2^The First People's Hospital of Baiyin, Baiyin 730900, China; ^3^School of Nursing, Gansu University of Chinese Medicine, Lanzhou 730000, China; ^4^PRIDE Research Foundation, 7929 Brookriver Dr. Ste. 400, Dallas, Texas 75247, USA

## Abstract

**Background:** Strong associations have been demonstrated between chronic musculoskeletal pain, pain-related fear-avoidance (FA) of activities of daily living, and functional disability. The Fear Avoidance Components Scale (FACS) is a patient-reported outcome (PRO) measure, which was designed to evaluate cognitive, emotional, and behavioural dimensions of FA.

**Objective:** The study aims were to translate the English version of the FACS into Simplified Chinese and then to examine its psychometric properties.

**Methods:** The translation and cross-cultural adaptation of the FACS from English to Chinese was performed with standard methodology. A group of 330 subjects with chronic musculoskeletal pain completed the FACS-Chi and additional FA-related PRO measures. The FACS-Chi was then completed a second time, 1 week later.

**Results:** The FACS-Chi showed excellent internal consistency (Cronbach's alpha = 0.920) and test-retest reliability (ICC = 0.918). A confirmatory factor analysis of the 2-factor model determined in the original English version of FACS revealed an acceptable fit. Strong correlations were found between FACS-Chi scores and other PRO measures of perceived level of disability, pain catastrophizing, and pain-related anxiety (*p* < 0.001 for all analyses).

**Conclusions:** The FACS-Chi demonstrated good psychometric properties, including excellent test-retest reliability and internal consistency and satisfactory construct validity. The FACS-Chi may be a useful measure of pain-related FA in Chinese-speaking patients with chronic musculoskeletal pain.

## 1. Introduction

Chronic pain is a common condition, affecting about 20% of the population worldwide [1]. The 2016 Global Burden of Disease Study reports that pain and pain-related disorders are the leading cause of disability and burden of disease for patients [2]. In middle- and low-income countries, the prevalence of chronic pain is 33% among adults and up to 56% among older persons [3]. Data show a high prevalence of chronic pain in the Chinese population, more than 30% among the adults and increasing to nearly 50% among older people [4, 5]. However, to our knowledge, pain treatment is currently less effective in clinical practice and there is a relative lack of high quality-research studies about the management of chronic pain in Chinese patients [6].

Mechanisms of pain are varied and complex, especially when acute pain develops into a chronic condition [7, 8]. The experience of chronic pain can involve a variety of sensory, emotional, cognitive and interoceptive components [8–11]. The Fear-avoidance (FA) Model of Chronic Pain provides one explanation for the transition from acute to chronic pain. It was originally proposed by Lethem et al. in 1983 [12] and further developed by Vlaeyen et al. in 1995 [13]. The current FA model incorporates physiological, psychological and sociological elements to describe how fear of pain and injury can lead to physical deconditioning, depression and disability from activities of daily living [14–16].

Several FA-related patient-reported outcome (PRO) questionnaires have been developed, including the Tampa Scale for Kinesiophobia (TSK) [17], the Pain Anxiety Symptom Scale (PASS) [18] the Pain Catastrophizing Scale (PCS) [19], and the Fear Avoidance Belief Questionnaire (FABQ) [20]. They have been widely used in many published clinical studies. However, because all four of these instruments were developed before the FA model was fully established, none of them provide a comprehensive assessment of the cognitive, emotional and behavioural components of the modern FA model of chronic pain [21]. In addition, some authors have raised concerns about some psychometric weaknesses (including limited construct validity and poorly-defined item specificity), the lack of well-defined cut-off values, and limited evidence of treatment responsiveness [22–24].

The Fear Avoidance Components Scale (FACS) was developed more recently in an attempt to overcome some of the deficiencies of previous FA-related PRO measures [25]. It has now been translated and psychometrically validated in multiple languages [26–31]. The FACS was designed to comprehensively evaluate FA beliefs and attitudes in persons with painful medical conditions. Some of the concepts from previous FA-related measures were incorporated in its construction. The original English version has demonstrated strong reliability, internal consistency, and content and constructs validity [25]. Two factors were initially identified in the English version based on exploratory factor analyses [32]: (1) general FA and (2) types of activities that are avoided [33]. A two-factor model has been confirmed in other translated versions of the FACS, though some slight differences in the results have been reported [26–29, 31]. Five severity levels have been recommended for clinical interpretation [25].

A comprehensive assessment of the patient's pain experience is vital to providing the most effective treatment. Chinese language versions of the TSK [34], PASS [35], and PCS [36] are currently available, with acceptable psychometric validations. However, a Chinese version of the FACS has not been previously published. A translated and culturally adapted Chinese version of the FACS could provide an additional tool for clinicians to assess pain-related fear and activity avoidance in Chinese-speaking patients. Hence, the goals of this study were to: (1) translate and cross-culturally adapt the English version of the FACS into Simplified Chinese and (2) evaluate its psychometric properties so that it can be made available to healthcare clinicians.

## 2. Materials and Methods

This study was done in two parts. In the first part, the FACS was translated from English to Chinese, followed by a backward translation and amendments for discrepancies. In the second part, a group of patients with chronic musculoskeletal pain completed the Chinese version of the FACS (FACS-Chi), along with some additional pain-related PRO measures, then repeated the FACS-Chi 1 week later. Following data collection, a psychometric assessment was performed.

### 2.1. FACS

The FACS is intended to comprehensively assess FA in patients with pain disorders [25]. The scale consists of 20 items, each of which is rated on a 6-point Likert scale, with scores ranging from 0 to 5 corresponding to “completely disagree,” “mostly disagree,” “somewhat disagree,” “somewhat agree,” “mostly agree,” and “completely agree,” respectively. The scale assesses 3 clinical dimensions of FA, including cognitive (8 entries), affective (6 entries) and behavioural (6 entries). The following severity levels have been recommended for clinical interpretation of total scores: subclinical (0–20), mild (21–40), moderate (41–60), severe (61–80) and extreme (81–100). Two factors were determined in the original English version. Factor 1 (including items 1–14) represents “general fear-avoidance” and Factor 2 (including Items 15–20) represent “types of activities that are avoided.” In the original published validation study, the FACS displayed excellent test-retest reliability with a Pearson correlation analysis of *r* between 0.90 and 0.94, as well as excellent internal consistency with a Cronbach's alpha of 0.92.

### 2.2. Subjects

Subjects were consecutively registered from January to July 2023 in a public hospital in Gansu. Inclusion criteria included: (a) aged 18–65 years; (b) Chinese nationality; (c) chronic musculoskeletal pain (pain symptoms lasting not less than 3 months) and (d) a signed informed consent form. Exclusion criteria included: (a) inability to complete the questionnaires due to deficits in mental or cognitive status; (b) diagnosis of certain diseases affecting the central nervous system, including cancer, brain or spinal cord injury, neurological diseases or injuries and (c) noncompliance or declined to participate in the experimental protocol. All participants were informed of the purpose of the study and were asked to give written consent before joining. The study was conducted in accordance with the Declaration of Helsinki and approved by the Ethics Committee of the Affiliated Hospital of Gansu University of Traditional Chinese Medicine ([2022]123).

### 2.3. Sample Size Calculation

Previous validation studies in other languages were used to calculate the sample size for this study [26]. A priori analysis, conducted with G Power 3.1, specified a minimum of 330 patients to detect a 0.2 effect size, with *α* = 0.05 and a power of 0.8 [37].

### 2.4. Translation Process

**Figure 1:**
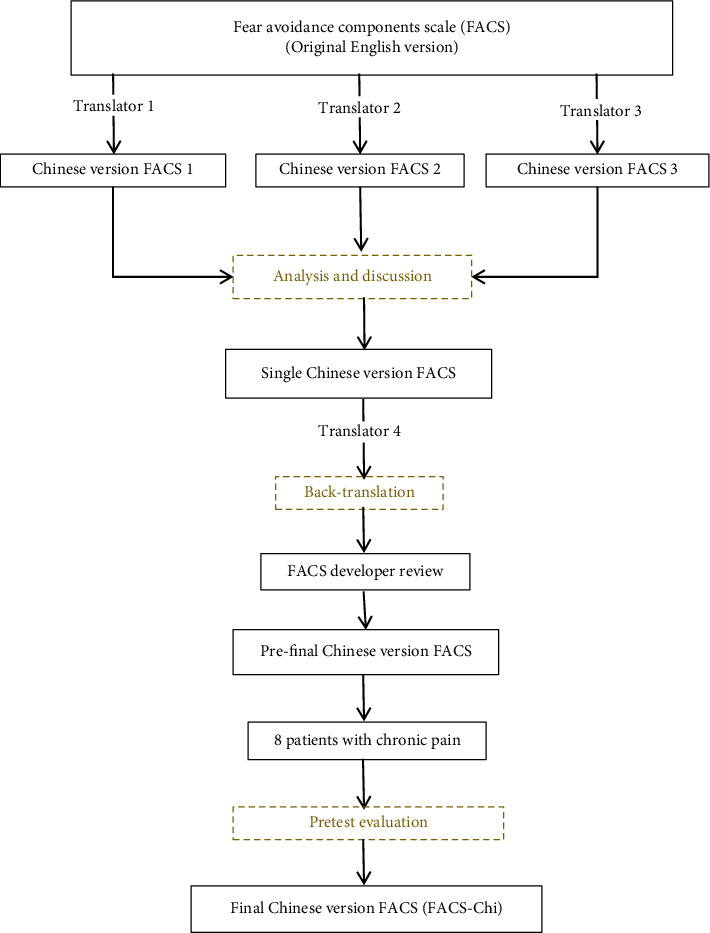
Translation process.

The translation and validation of the FACS for cross-cultural use followed the guidelines recommended by the International Society for Pharmacoeconomics and Outcomes Research (ISPOR) [38]. Translation from English to Chinese was completed by author ZT and two other medical experts. Each translator had studied in English-speaking regions. The translation process was conducted independently by each translator, without knowledge of the other participants, resulting in three Chinese versions of the FACS (FACS-Chi 1, FACS-Chi 2, FACS-Chi 3). After discussion, a single version of the FACS-Chi was agreement upon. A Chinese-English backward translation was then performed by a professional Chinese to English translator who had no prior exposure to the original English version of the FACS. An original FACS developer (author RN), who is also a native English speaker, participated in the review process of the backward translation to ensure the conceptual equivalence of the FACS-Chi translation to the English version. A pretesting process was then conducted on the prefinal translated version of the FACS-Chi with a sample of 8 patients, who were experiencing chronic musculoskeletal pain, to evaluate their comprehension of the items. The same inclusion and exclusion criteria were used as with the psychometric validation study patients, but these 8 patients were only selected for the pretest assessment. Based on the patient feedback in the pretest phase, no changes to the prefinal translated version were required. Therefore, the final version of FACS-Chi was generated and can be found in S1 Appendix. A flowchart of the translation process is shown in Figure 1. A PDF of the FACS-Chi, and other translated versions of the FACS, can also be found at https://www.pridedallas.com/questionnaires.

### 2.5. Patient-Reported Clinical Variables

In addition to the FACS-Chi, three other PRO measures were administered to assess psychosocial variables related to FA: the Oswestry Disability Index (ODI) [39], PCS [19] and PASS [18]. The Chinese versions adapted from these three original instruments have shown acceptable psychometric properties [35, 36, 40]. The ODI measures perceived disability with activities of daily living. It consists of 10 items with 6 options for each question, with a score range of 0 to 5 for each item and a total score range of 0 to 50. Higher scores reflect a higher level of disability. The PCS measures catastrophic thinking associated with the experience of pain. It contains 13 items rated on a 5-point Likert scale from 0 (not at all) to 4 (all the time). The total score ranges from 0 to 52. Higher scores reflect a higher level of pain-related catastrophizing. Three PCS subscales are also available: rumination, magnification, and helplessness. The PASS measures pain-related anxiety. Each item is rated on a 6-point Likert scale from 0 (“never”) to 5 (“frequently”) with a total score range of 0 to 100. Higher scores reflect a higher level of pain-related anxiety. Four subscales are available, including cognitive, avoidance, fear, and physiological anxiety, each containing five items.

### 2.6. Statistical Analyses

Descriptive statistical analysis was used to determine means and standard deviations of demographic variables. Differences between patient subgroups and sample distribution and normality (including skewness, kurtosis and histograms) were assessed with Analysis of Variance (ANOVA) or Kruskal–Wallis tests, depending on the distribution. If the assumption of homogeneity of variance was confirmed, an ANOVA with post hoc Tukey's test was used. Otherwise, the Kruskal–Wallis test was used. To determine whether there were differences in the distributions of categorical variables, a chi-squared test was used. Data analyses were performed using the IBM SPSS Statistics 26 (IBM Corp.; NY; Armonk) and IBM SPSS Amos 26 Graphics (IBM Corp.; NY; USA).

Test-retest reliability of the FACS-Chi was calculated by intraclass correlation coefficients type 2, 1 (ICC 2, 1). ICC 0 to 0.5 is considered poor, 0.5 to 0.75 is considered moderate, 0.75 to 0.9 is considered good, and 0.9 to 1 is considered excellent [41]. The Cronbach's alpha (*α*) coefficient was used to estimate internal consistency. *α* > 0.7 is considered acceptable; *α* > 0.8 is considered high; and *α* > 0.9 is considered excellent ADDIN NE.Ref.{1B09F4A9-9EDC-4086-B3D9-1B7B927BB937} Confirmatory factor analysis (CFA) for ordinal data were used for construct validity of FACS-Chi and to test the original 2 factor model suggested by Neblett et al. [25]. The model fit was assessed using the following indicators: Chi-square/degree of freedom (*χ*^2^/*df* < 5, considered acceptable), root mean square error of approximation (RMSEA ≤ 0.08, considered fair), comparative fit index (CFI > 0.9, considered good), Tucker–Lewis index (TLI > 0.9, considered good), Incremental Fit Index (IFI > 0.9, considered good), and Normed Fit Index (NFI > 0.9, considered good) [42]. Correlations among the FACS-Chi, ODI, PCS, and PASS items were assessed for convergent validity. PASS scores did not conform to a normal distribution, so Spearman's correlation coefficient (*r*) was used to assess the strength of the correlation with *r* > 0.3 determined to be moderate.

## 3. Results

### 3.1. Subject Characteristics

Characteristics of the study subjects can be found in Table 1. Slightly more than half of the subjects were male (182/330, 55.2%) with a mean age of 51.35 ± 10.62 years. The majority of the subjects reported chronic spinal pain (291/330, 88%). Only 12% (39/330) reported chronic pain in one or more limbs without any spinal pain. Pain duration ranged from 3 months to over 12 years. The highest percentage of subjects reported a pain duration of 3–6 months (234/330, 70.9%).

### 3.2. Assessment of Test-Retest Reliability and Internal Consistency

FACS-Chi reliability results can be found in Table 2. Excellent test-retest reliability was found for the total score (ICC2, 1 = 0.918), Factor 1 (ICC2, 1 = 0.923) and Factor 2 (ICC2, 1 = 0.891). The internal consistency was excellent (0.920), with Cronbach's alpha for Factor 1 = 0.898, and Cronbach's alpha for Factor 2 = 0.817.

### 3.3. Assessment of Construct Validity

**Figure 2:**
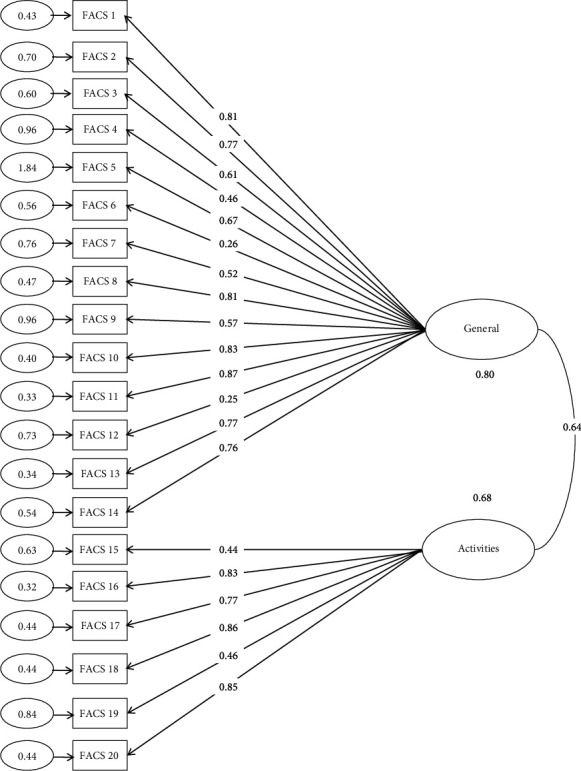
Factor loadings and goodness of fit indexes for underlying two factors of the FACS-Chi. *χ*^2^ = 354.626, *df* = 110, *p* < 0.001, RMSEA = 0.082.

The CFA results showed an acceptable fit: *χ*^2^ = 354.626, *df* = 110, *p* < 0.001, CFI = 0.946, TLI = 0.907, IFI = 0.947, NFI = 0.907, RMSEA = 0.082. All indicators were within the recommended ranges, indicating a good model fit. The model fit indicators are shown in Table 3 and Figure 2.

### 3.4. Assessment of Convergent Validity

Correlational results can be found in Table 4. The correlations among the FACS-Chi, Factor 1, Factor 2 and other indicators, including the ODI, PCS and PASS were all statistically significant at the 0.01 level (1-tailed).

### 3.5. Assessment of Severity Subgroups

**Figure 3:**
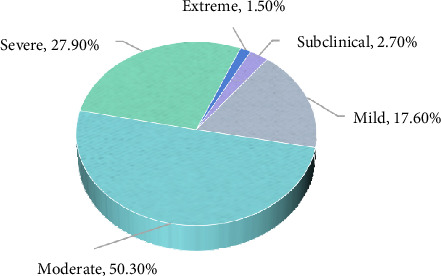
Distribution of severity levels of the FACS-Chi.

Subjects were grouped into FACS-Chi severity levels for additional analyses. Distribution of FACS-Chi severity subgroups can be found in Figure 3. Approximately 50% (166/330) of the participants scored in a moderate range and 27.9% (92/330) scored in a severe range. Very few scored in the subclinical (9/330; 2.7%) or extreme (5/330; 1.5%) severity ranges.

The relationship among FACS-Chi severity subgroup mean scores and the other PRO measures are shown in Table 5. Mean scores on all the PRO measures increased in a “stair-step” fashion from subclinical to severe FACS-Chi subgroups. Significant differences were found for all variables, including ODI, PCS, PASS and PASS subscale scores (*p* < 0.001). Significant differences were also found for most comparisons among individual subgroup scores. The FACS-Chi severity subgroups did not differ significantly in terms of gender (*χ*^2^ = 4.366, *p*=0.359), age (Kruskal–Wallis *χ*^2^ = 0.697, *p*=0.952), BMI (Kruskal–Wallis *χ*^2^ = 0.179, *p*=0.996), or duration of pain (Kruskal–Wallis *χ*^2^ = 5.377, *p*=0.251), but did differ in terms of pain location (Kruskal–Wallis *χ*^2^ = 17.746, *p*=0.001).

## 4. Discussion

The original English version of the FACS was successfully translated and culturally adapted into Simplified Chinese, and the new FACS-Chi version was psychometrically validated. The FACS-Chi demonstrated excellent test-retest reliability and internal consistency. These results are comparable to other FACS versions, including English [25], Serbian [26], French [27], Turkish [31], Spanish [28], Dutch [29] and Gujarati [30].

The CFA test for the FACS-Chi determined an acceptable fit with the original 2-factor model proposed by Neblett et al. [25]. This is similar to the CFA results of the Serbian version [26]. Notably, the average variance extracted (AVE) for Factor 1 in this study was 0.4244 < 0.5, whereas the composition reliability value (CR) was 0.9032. Although the general standard requires that the AVE is more than 0.5, the model is still considered acceptable, according to the suggestion by Fornell and Larcker [43]. They noted that if AVE is less than 0.5, but higher than 0.4, and CR is higher than 0.6, the convergent validity of the construct is still adequate [44].

Previous studies have reported associations between FACS scores and FA-related depressive symptoms, perceived disability, perceived injustice, insomnia, and kinesiophobia [25, 33]. Our findings support these previous results. The FACS-Chi was positively correlated with the ODI, PCS, and PASS. All correlations were significant at the 0.01 level (1-tailed). It should be noted that validated Chinese versions of the ODI, PCS and PASS [35, 36, 40] were used in our study to reduce bias due to cultural differences.

The subjects were categorized into five FA severity level subgroups based on total FACS-Chi scores [25]. The distribution of patients was centrally concentrated in the moderate (50.3%) and severe (27.9%) subgroups, with few patients in the subclinical (2.7%) and extreme (1.5%) subgroups. In comparison to our results, 67.5% of patients in the original English study [25], 50.7% in the Gujarati studies [30], 46.0% in the Serbian study [26], and 39% in the Dutch study [29] scored in the severe to extreme ranges. These differences in FACS severity level distributions are likely be due to differences in subject characteristics, including presenting medical disorders, pain duration and cultural influences. It was noted that a large number of participants in our study reported pain within the previous 3–6 months (234/330, 70.9%), whereas the average duration of disability was 30.9 months in the original English study [33], and 26.3 months in the Dutch study [29]. Some previous studies have found positive correlations between FA beliefs and pain duration [45–47], which may help explain the higher FACS scores found in some previous studies. Unfortunately, not all previous FACS studies have reported pain duration of their study subjects, so a comprehensive assessment of the relationship between pain duration and FACS scores is not currently available. It was also noted that relatively few subjects scored within the subclinical FACS-Chi severity range. An ethnic factor may help explain this result. Previous studies have found that Chinese subjects reported greater pain catastrophizing and lower pain tolerance than European Canadians in a cold-pressor pain test [48] and were more sensitive to mechanical deep pain and mechanical pain rating parameters than Danes [49]. Chinese cultural influences may provide some explanation for the large percentage of subjects who scored in the moderate FACS-Chi severity range. In Chinese culture, the “the Doctrine of the Mean” or “the Way of Balance” advocates for a balanced approach to life, avoiding extremes and excesses [50].

To further assess the validity of the FACS-Chi, comparisons of ODI, PCS and PASS scores (including PCS and PASS subscale scores) were made for each FAC-Chi severity subgroup. Scores on all the PRO measures increased in a “stair-step” pattern with each increase in FACS-Chi severity, from subclinical to extreme. Significant differences were found between all the variables, with large effect sizes. These results are generally consistent with those of the Serbian FACS study, in which a similar analysis was performed. Significant differences in PRO scores were also found among most of the individual group comparisons. However, no significant score differences were found between Subclinical and Mild FACS-Chi severity groups for any of the other PRO measures. Interestingly, the Serbian study did find significant ODI and PCS total score differences between the FACS subclinical and mild subgroups. Our results indicate that the cognitive, emotional, and behavioural function of chronic pain patients could possibly be affected by FA beliefs, particularly when those beliefs reach a certain severity level.

A variety of studies have found a strong relationship between perceived functional disability and pain related fear, anxiety and depression [33, 51–53]. Because of the complex nature of chronic pain, and the variety of psychosocial factors that can impact one's pain experience, current research has advocated the importance of a biopsychosocial approach in chronic pain management [54, 55]. In addition, Cognitive Functional Therapy (CFT) has shown good efficacy in improving pain and disability [56]. CFT is a patient-centered approach that helps patients self-manage their pain by targeting the individual pain-related cognitions, emotions and behaviours. The FACS-Chi may prove to be a useful clinical tool for measuring pain related beliefs and activity avoidance in Chinese-speaking patients with chronic musculoskeletal pain. The addition of the FACS-Chi to a standard PRO test battery may provide additional clinical information to help with treatment planning to address FA-related cognitive, emotional and behavioural barriers to function with activities of daily living.

### 4.1. Strengths and Limitations

There were some strengths in this study. The translation and cultural adaptation process rigidly followed guidelines recommended by the ISPOR [38]. No subjects dropped out of the study. All subjects completed the FACS-Chi and the other patient-reported measures, which may have minimized reporting biases. Finally, the sample size was sufficient to support subgroup analyses.

There were also some limitations in this study. First, as mentioned above, the sample distribution was concentrated in the middle FAC-Chi severity ranges, with few participants in the subclinical and extreme groups. Second, our study enrolled 330 patients with chronic musculoskeletal pain from a single centre, the Outpatient Department of Rehabilitation Medicine of the Affiliated Hospital of Gansu University of Traditional Chinese Medicine, which limited the variety of the chronic pain conditions of the patients enrolled in our study and the generalisability of the results. Finally, the sample size limited our ability to conduct exploratory factor analyses of the FACS-Chi. Therefore, we based our results on exploratory factor analyses from previous studies.

## 5. Conclusion

In conclusion, the FACS-Chi demonstrated excellent internal consistency and test-retest reliability and satisfactory construct validity. Therefore, it can be used to measure pain-related fear and avoidance in Chinese-speaking patients with chronic musculoskeletal pain.

## Figures and Tables

**Table 1:** Subject characteristics (*n* = 330).

Item	Mean ± SD/*n* (%)
*Age (year)*	
Male	51.84 ± 14.48
Female	52.84 ± 15.96

*Gender*	
Male	182 (55.2%)
Female	148 (44.8%)

*Height (cm)*	
Male	172.46 ± 2.90
Female	159.68 ± 5.01

*Weight (kg)*	
Male	75.05 ± 5.30
Female	64.74 ± 4.91

*BMI*	
Male	25.26 ± 2.02
Female	25.40 ± 1.83

*Pain site*	
Cervical spine	76 (23.0%)
Lumbar spine	159 (48.2%)
Multiple areas of the spine	41 (12.4%)
One or more limbs	39 (11.8%)
Spine with other body parts	15 (4.5%)

*Duration of pain (months)*	
3–6	234 (70.9%)
7–9	61 (18.5%)
10–12	30 (9.1%)
> 12	5 (1.5%)

**Table 2:** Intraclass correlation coefficients and 95% confidence interval (lower-upper bound) of test-retest reliability.

Item	Intraclass correlation coefficients	95% confidence interval (lower-upper bound)
1	0.905	0.884∼0.923
2	0.909	0.888∼0.926
3	0.886	0.859∼0.907
4	0.911	0.890∼0.928
5	0.920	0.901∼0.935
6	0.948	0.935∼0.958
7	0.789	0.738∼0.831
8	0.852	0.819∼0.879
9	0.870	0.839∼0.896
10	0.918	0.899∼0.934
11	0.889	0.860∼0.911
12	0.876	0.847∼0.900
13	0.836	0.796∼0.868
14	0.879	0.850∼0.902
15	0.820	0.781∼0.853
16	0.777	0.729∼0.817
17	0.880	0.851∼0.904
18	0.843	0.808∼0.872
19	0.780	0.733∼0.819
20	0.830	0.793∼0.861
Factor 1^a^	0.923	0.903∼0.939
Factor 2^b^	0.891	0.866∼0.911
FACS-chi total score	0.918	0.897∼0.934

^a^“General Fear Avoidance”.

^b^“Types of Activities that are Avoided”.

**Table 3:** The average variance extracted (AVE) and combined reliability (CR) of the FACS-Chi.

Item		Estimate	AVE	CR
Factor 1	1	0.806	0.4244	0.9032
	2	0.765		
	3	0.607		
	4	0.457		
	5	0.670		
	6	0.262		
	7	0.522		
	8	0.812		
	9	0.566		
	10	0.873		
	11	0.246		
	12	0.775		
	13	0.853		
	14	0.457		

Factor 2	15	0.862	0.5804	0.889
	16	0.772		
	17	0.825		
	18	0.760		
	19	0.831		
	20	0.441		

**Table 4:** Correlations among patient-reported variables.

	FACS	ODI	PCS	PASS	Factor 1	Factor 2
FACS	1.000					
ODI	0.427	1.000				
PCS	0.697	0.494	1.000			
PASS	0.474∗	0.482∗	0.504∗	1.000		
Factor 1	0.945	0.395	0.657	0.429∗	1.000	
Factor 2	0.824	0.374	0.600	0.438∗	0.713	1.000

*Note:* All correlations were significant at the 0.01 level (1-tailed).

^∗^Spearman's correlation.

**Table 5:** Patient-reported clinical variables by FACS-Chi severity groups.

Patient reported variables mean (SD)	Total (0–100)	FACS-Chi severity groups					*χ* ^2^/*F*	*p*	Effect size (partial eta squared)^g^
		Subclinical (0–20)^a^	Mild (21–40)^b^	Moderate (41–60)^c^	Severe (61–80)^d^	Extreme (81–100)^e^			
*N* (%)	330 (100)	9 (2.7)	58 (17.6)	166 (50.3)	92 (27.9)	5 (1.5)			
FACS	52.43 (14.13)	16.56 (4.13)^bcde^	34.03 (5.27)^acde^	51.57 (5.52)^abde^	67.38 (4.83)^abce^	83.80 (2.17)^abcd^	*F* = 502.995	< 0.001	0.861
ODI	26.45 (8.48)	19.33 (10.37)^cde^	21.02 (7.88)^cde^	26.42 (7.03)^abde^	29.67 (8.34)^abce^	44.2 (4.27)^abcd^	*F* = 20.365	< 0.001	0.200
PCS total score	25.96 (7.64)	17.33 (7.50)^cde^	18.07 (5.84)^cde^	25.46 (5.65)^abde^	31.73 (5.20)^abce^	43.60 (5.55)^abcd^	*F* = 69.246	< 0.001	0.460
PCS rumination subscale	11.64 (3.83)	7.11 (3.55)^cde^	8.41 (2.74)^cde^	11.29 (3.01)^abde^	14.38 (3.29)^abce^	19.20 (3.35)^abcd^	*F* = 47.403	< 0.001	0.330
PCS magnification subscale	8.27 (2.76)	5.67 (2.35)^cde^	5.69 (2.74)^cde^	8.22 (2.25)^abde^	9.99 (1.92)^abce^	13.40 (1.52)^abcd^	*F* = 41.573	< 0.001	0.365
PCS helplessness subscale	6.05 (2.14)	4.56 (2.35)^cde^	3.97 (1.70)^cde^	5.97 (1.66)^abde^	7.40 (1.73)^abce^	11.00 (1.22)^abcd^	*F* = 48.371	< 0.001	0.361
PASS total score	46.65 (14.68)	31.56 (9.70)^cde^	34.24 (13.64)^cde^	47.78 (11.61)^abe^	52.85 (15.04)^ab^	66.00 (5.10)^abcd^	*χ* ^2^ = 77.759^f^	< 0.001	—
PASS cognition subscale	12.48 (4.06)	6.33 (0.87)^cde^	9.59 (3.30)^cde^	12.32 (3.10)^abde^	14.83 (4.21)^abc^	19.60 (1.34)^abc^	*χ* ^2^ = 94.135^f^	< 0.001	—
PASS avoidance subscale	10.80 (3.98)	6.89 (2.71)^cde^	7.93 (3.81)^cde^	11.08 (3.20)^abe^	12.20 (4.26)^ab^	16.40 (2.30)^abc^	*χ* ^2^ = 64.921^f^	< 0.001	—
PASS fear subscale	12.10 (4.47)	10.44 (4.33)	8.91 (4.11)^cde^	12.70 (4.04)^b^	13.01 (4.62)^b^	15.00 (2.24)^b^	*χ* ^2^ = 40.211^f^	< 0.001	—
PASS physiological anxiety subscale	15.00 (4.85)	7.89 (5.06)^cde^	7.81 (4.75)^cde^	11.67 (5.21)^ab^	12.49 (6.00)^ab^	15.00 (4.85)^ab^	*F* = 8.496	< 0.001	0.095

*Note:* ^a,b,c,d,e^Groups (different symbols) that significantly differed from each other in continuous comparisons. ^f^The Kruskal–Wallis test was applied, and the *χ*^2^ value is reported because the assumption of homogeneity of variances was not met for this variable. ^g^Effect size (partial eta squared): 0.01 = small; 0.06 = medium; 0.14 = large.

## Data Availability

The data that support the findings of this study are available on request from the corresponding author. The data are not publicly available due to privacy or ethical restrictions.

## Supporting Information

The Supporting Information file name is “S1 Appendix FACS-chi.pdf,” which is the Chinese version of the Fear Avoidance Components Scale (FACS). This is the Chinese version of the Fear Avoidance Components Scale (FACS). The FACS is used to assess an individual's response to chronic pain, in particular their feelings and thoughts about pain and the impact of pain on their daily activities. The scale is used in clinical or research settings to help healthcare professionals understand the psychological states and behavioural patterns of people with chronic pain in order to better plan treatment.

## References

[1] Treede R. D., Rief W., Barke A. (2015). A Classification of Chronic Pain for ICD-11. *Pain*.

[2] Jiang Y., Xu T., Mao F. (2022). The Prevalence and Management of Chronic Pain in the Chinese Population: Findings from the China Pain Health Index. *Population Health Metrics*.

[3] Jackson T., Thomas S., Stabile V., Han X., Shotwell M., McQueen K. (2015). Prevalence of Chronic Pain in Low-Income and Middle-Income Countries: a Systematic Review and Meta-Analysis. *The Lancet*.

[4] Li J., Chen J., Qin Q. (2018). Chronic Pain and its Association with Obesity Among Older Adults in China. *Archives of Gerontology and Geriatrics*.

[5] Yongjun Z., Tingjie Z., Xiaoqiu Y. (2020). A Survey of Chronic Pain in China. *Libyan Journal of Medicine*.

[6] Liu W., Luo A., Liu H. (2007). Overcoming the Barriers in Pain Control: An Update of Pain Management in China. *European Journal of Pain Supplements*.

[7] Fornasari D. (2012). Pain Mechanisms in Patients With Chronic Pain. *Clinical Drug Investigation*.

[8] Kuner R., Flor H. (2017). Erratum: Structural Plasticity and Reorganisation in Chronic Pain. *Nature Reviews Neuroscience*.

[9] Simons L. E., Elman I., Borsook D. (2014). Psychological Processing in Chronic Pain: a Neural Systems Approach. *Neuroscience & Biobehavioral Reviews*.

[10] Atlas L. Y., Al’Absi M. (2018). The Neuroscience of Pain: Biobehavioral, Developmental, and Psychosocial Mechanisms Relevant to Intervention Targets. *Psychosomatic Medicine*.

[11] Widerström-Noga E. G., Finnerup N. B., Siddall P. J. (2009). Biopsychosocial Perspective on a Mechanisms-Based Approach to Assessment and Treatment of Pain Following Spinal Cord Injury. *Journal of Rehabilitation Research and Development*.

[12] Lethem J., Slade P. D., Troup J. D., Bentley G. (1983). Outline of a Fear-Avoidance Model of Exaggerated Pain Perception-I. *Behaviour Research and Therapy*.

[13] Vlaeyen J., Kole-Snijders A., Boeren R., van Eek H. (1995). Fear of Movement/(re)injury in Chronic Low Back Pain and Its Relation to Behavioral Performance. *Pain*.

[14] Crombez G., Eccleston C., Van Damme S., Vlaeyen J. W., Karoly P. (2012). Fear-avoidance Model of Chronic Pain: the Next Generation. *The Clinical Journal of Pain*.

[15] Zhao X., Boersma K., Gerdle B., Molander P., Hesser H. (2023). Fear Network and Pain Extent: Interplays Among Psychological Constructs Related to the Fear-Avoidance Model. *Journal of Psychosomatic Research*.

[16] González Aroca J., Díaz Á. P., Navarrete C., Albarnez L. (2023). Fear-Avoidance Beliefs Are Associated With Pain Intensity and Shoulder Disability in Adults with Chronic Shoulder Pain: A Cross-Sectional Study. *Journal of Clinical Medicine*.

[17] Weermeijer J. D., Meulders A. (2018). Clinimetrics: Tampa Scale for Kinesiophobia. *Journal of Physiotherapy*.

[18] McCracken L. M., Zayfert C., Gross R. T. (1992). The Pain Anxiety Symptoms Scale: Development and Validation of a Scale to Measure Fear of Pain. *Pain*.

[19] Sullivan M., Bishop S., Pivik J. (1995). The Pain Catastrophizing Scale: Development and Validation. *Psychological Assessment*.

[20] Waddell G., Newton M., Henderson I., Somerville D., Main C. J. (1993). A Fear-Avoidance Beliefs Questionnaire (FABQ) and the Role of Fear-Avoidance Beliefs in Chronic Low Back Pain and Disability. *Pain*.

[21] Client X. N. A. (2021). *There Are More than 300 Million Patients with Chronic Pain in China, and Pain Has Become the Third Major Health Problem*.

[22] Pincus T., Smeets R. J., Simmonds M. J., Sullivan M. J. (2010). The Fear Avoidance Model Disentangled: Improving the Clinical Utility of the Fear Avoidance Model. *The Clinical Journal of Pain*.

[23] Lundberg M., Grimby-Ekman A., Verbunt J., Simmonds M. J. (2011). Pain-related Fear: A Critical Review of the Related Measures. *Pain Research and Treatment*.

[24] Rainville J., Smeets R. J., Bendix T., Tveito T. H., Poiraudeau S., Indahl A. J. (2011). Fear-Avoidance Beliefs and Pain Avoidance in Low Back Pain-Translating Research into Clinical Practice. *The Spine Journal*.

[25] Neblett R., Mayer T. G., Hartzell M. M., Williams M. J., Gatchel R. J. (2016). The Fear-Avoidance Components Scale (FACS): Development and Psychometric Evaluation of a New Measure of Pain-Related Fear Avoidance. *Pain Practice*.

[26] Knezevic A., Neblett R., Gatchel R. J. (2018). Psychometric Validation of the Serbian Version of the Fear Avoidance Component Scale (FACS). *PLoS One*.

[27] Duport A., Sonia B., Catherine R. (2023). Cross-Cultural Translation and Psychometric Validation of the French Version of the Fear-Avoidance Components Scale (FACS). *medRxiv*.

[28] Cuesta-Vargas A. I., Neblett R., Gatchel R. J., Roldán-Jiménez C. (2020). Cross-cultural Adaptation and Validity of the Spanish Fear-Avoidance Components Scale and Clinical Implications in Primary Care. *BMC Family Practice*.

[29] De Baets L., Sergooris A., Neblett R. (2023). The Development and Measurement Properties of the Dutch Version of the Fear-Avoidance Components Scale (FACS-D) in Persons With Chronic Musculoskeletal Pain. *Scandinavian Journal of Pain*.

[30] Bid D. D., Neblett R., Alagappan T. R. (2020). Cross-Cultural Adaptation, Reliability, and Validity of the Gujarati Fear-Avoidance Components Scale. *Physiotherapy-The Journal of Indian Association of Physiotherapists*.

[31] Turan K., Sarı Z., Özden F. (2023). Psychometric Properties of the Turkish Version of the Fear Avoidance Components Scale in Patients with Chronic Pain Related to Musculoskeletal Disorders. *Wiener Klinische Wochenschrift*.

[32] Orçan F. (2018). Exploratory and Confirmatory Factor Analysis: Which One to Use First?. *Eğitimde ve Psikolojide Ölçme ve Değerlendirme Dergisi*.

[33] Neblett R., Mayer T. G., Williams M. J. (2017). The Fear-Avoidance Components Scale (FACS): Responsiveness to Functional Restoration Treatment in a Chronic Musculoskeletal Pain Disorder (CMPD) Population. *The Clinical Journal of Pain*.

[34] Wei X., Xu X., Zhao Y., Hu W., Bai Y., Li M. (2015). The Chinese Version of the Tampa Scale for Kinesiophobia Was Cross-Culturally Adapted and Validated in Patients With Low Back Pain. *Journal of Clinical Epidemiology*.

[35] Zhou X. Y., Xu X. M., Wang F. (2017). Validations and Psychological Properties of a Simplified Chinese Version of Pain Anxiety Symptoms Scale (SC-PASS). *Medicine (Baltimore)*.

[36] Shen B., Wu B., Abdullah T. B. (2018). Translation and Validation of Simplified Chinese Version of the Pain Catastrophizing Scale in Chronic Pain Patients: Education May Matter. *Molecular Pain*.

[37] Faul F., Erdfelder E., Lang A. G., Buchner A. (2007). G^∗^Power 3: a Flexible Statistical Power Analysis Program for the Social, Behavioral, and Biomedical Sciences. *Behavior Research Methods*.

[38] Wild D., Grove A., Martin M. (2005). Principles of Good Practice for the Translation and Cultural Adaptation Process for Patient-Reported Outcomes (PRO) Measures: Report of the ISPOR Task Force for Translation and Cultural Adaptation. *Value in Health*.

[39] Fairbank J. C., Pynsent P. B. (2000). The Oswestry Disability Index. *Spine*.

[40] Liu H., Tao H., Luo Z. (2009). Validation of the Simplified Chinese Version of the Oswestry Disability Index. *Spine*.

[41] Deyo R. A., Diehr P., Patrick D. L. (1991). Reproducibility and Responsiveness of Health Status Measures Statistics and Strategies for Evaluation. *Controlled Clinical Trials*.

[42] Wolf M. G., McNeish D. (2023). Dynamic: An R Package for Deriving Dynamic Fit Index Cutoffs for Factor Analysis. *Multivariate Behavioral Research*.

[43] Fornell C., Larcker D. F. (1981). Evaluating Structural Equation Models With Unobservable Variables and Measurement Error. *Journal of Marketing Research*.

[44] Lam L. W. (2012). Impact of Competitiveness on Salespeople’s Commitment and Performance. *Journal of Business Research*.

[45] Hida M., Nakamura M., Imaoka M. (2020). Effects of the Characteristics and Duration of Chronic Pain on Psychosomatic Function in the Community-Dwelling Elderly Population. *Pain Research and Management*.

[46] Sanson N., Hach S., Moran R., Mason J. (2020). Behavioural Activation and Inhibition Systems in Relation to Pain Intensity and Duration in a Sample of People Experiencing Chronic Musculoskeletal Pain. *Musculoskeletal Science and Practice*.

[47] Severeijns R., Vlaeyen J. W., van den Hout M. A., Weber W. E. (2001). Pain Catastrophizing Predicts Pain Intensity, Disability, and Psychological Distress Independent of the Level of Physical Impairment. *The Clinical Journal of Pain*.

[48] Campbell C. M., Edwards R. R. (2012). Ethnic Differences in Pain and Pain Management. *Pain Management*.

[49] Yang G., Luo Y., Baad Hansen L. (2013). Ethnic Differences in Oro‐facial Somatosensory Profiles—Quantitative Sensory Testing in C Hinese and D Anes. *Journal of Oral Rehabilitation*.

[50] Gao R., Huang S., Yao Y. (2022). Understanding Zhongyong Using a Zhongyong Approach: Re-examining the Non-linear Relationship Between Creativity and the Confucian Doctrine of the Mean. *Frontiers in Psychology*.

[51] Pwm M., Schabrun S., Knox M. F. (2017). Physical Activity and the Mediating Effect of Fear, Depression, Anxiety, and Catastrophizing on Pain Related Disability in People With Chronic Low Back Pain. *PLoS One*.

[52] Lerman S. F., Rudich Z., Brill S., Shalev H., Shahar G. (2015). Longitudinal Associations Between Depression, Anxiety, Pain, and Pain-Related Disability in Chronic Pain Patients. *Psychosomatic Medicine*.

[53] Gheldof E. L., Crombez G., Van den Bussche E. (2010). Pain-related Fear Predicts Disability, But Not Pain Severity: a Path Analytic Approach of the Fear-Avoidance Model. *European Journal of Pain*.

[54] Meeus M., Nijs J., Wilgen P. V., Noten S., Huijnen I. (2016). Moving on to Movement in Patients With Chronic Joint Pain.

[55] Bernstein I. A., Malik Q., Carville S., Ward S. (2017). Low Back Pain and Sciatica: Summary of NICE Guidance. *BMJ*.

[56] Kent P., Haines T., O’Sullivan P. (2023). Cognitive Functional Therapy With or Without Movement Sensor Biofeedback Versus Usual Care for Chronic, Disabling Low Back Pain (RESTORE): A Randomised, Controlled, Three-Arm, Parallel Group, Phase 3, Clinical Trial. *Lancet*.

