# Supplementary material for: Cultural Adaptation and Psychometric Validation of the Simplified Chinese Version of the Fear Avoidance Component Scale (FACS)
Source: Pain Res Manag. 2024 Dec 5;2024:7966689. doi: 10.1155/prm/7966689 (PMC11637624; doi:10.1155/prm/7966689)
Supplement: Supporting Information — Additional supporting information can be found online in the Supporting Information section. [file 7966689.f1.pdf]

恐惧回避综合量表

姓名：______________　　　　日期：____/____/________

说明：每个人对疼痛的反应不同。我们希望知晓：您对自己病痛有怎样的感受和想法；这些病痛给您的活动造成了多大的影响。请仔细思考您在过去一周的经历，并在下面量表的每项中，圈出“0”到“5”之间的一个数字回答问题。

5 = 完全同意

4 = 大部分同意

3 = 较为同意

2 = 较为不同意

1 = 大部分不同意

0 = 完全不同意

**在过去的一周内，您对这些描述您病痛的陈述有何种程度的赞同/不赞同？**	完全同意	大部分同意	较为同意	较为不同意	大部分不同意	完全不同意
1. 我会避免加重疼痛的活动和动作	5	4	3	2	1	0
2. 我对我的病痛感到担忧	5	4	3	2	1	0
3. 我认为我的疼痛会持续恶化直到我完全无法活动	5	4	3	2	1	0
4. 当我想起我的病痛，恐惧使我无法承受	5	4	3	2	1	0
5. 我不会去尝试某些活动，因为我担心这会让我受伤（或再次受伤）	5	4	3	2	1	0
6. 当剧烈疼痛时，我随之出现了诸如恶心，呼吸困难，心跳加速，发抖，以及/或头晕的症状	5	4	3	2	1	0
7. 不得不忍受自己的病痛让我觉得不公平	5	4	3	2	1	0
8. 我的病痛让我今后处于受伤（或再次受伤）的风险中	5	4	3	2	1	0

继续……

**在过去的一周内，您对这些描述您病痛的陈述有何种程度的赞同/不赞同？**	完全同意	大部分同意	较为同意	较为不同意	大部分不同意	完全不同意
9. 因为我的病痛，我的生活不会和以前一样	5	4	3	2	1	0
10. 我无法控制我的疼痛	5	4	3	2	1	0
11. 我不会去尝试某些活动和动作，因为我担心这会让我疼痛加剧	5	4	3	2	1	0
12. 我的病痛是别人造成的	5	4	3	2	1	0
13. 病症产生的疼痛是我身体某部分出现严重问题的警示信号	5	4	3	2	1	0
14. 没人理解我的病痛有多严重	5	4	3	2	1	0

**请以这句话为开头完成对以下每一项的回答：在过去的一周里，由于我的病痛，我会避免（以下情况）……**	完全同意	大部分同意	较为同意	较为不同意	大部分不同意	完全不同意
15. ……费力的活动（例如繁重的庭院活动或搬重的家具）	5	4	3	2	1	0
16. ……中等程度的活动（例如烹饪或做清洁）	5	4	3	2	1	0
17. ……轻体力活动（例如去看电影或外出吃午餐）	5	4	3	2	1	0
18. ……我全职的家务杂事/或工作事务	5	4	3	2	1	0
19. ……娱乐和/或运动（让我开心和对健康有益的事情）	5	4	3	2	1	0
20. ……不得不需要让我身体疼痛部位参与的活动	5	4	3	2	1	0

总分：______________
